# Associations between rs3480 and rs16835198 gene polymorphisms of *FNDC5* with type 2 diabetes mellitus susceptibility: a meta-analysis

**DOI:** 10.3389/fendo.2022.946982

**Published:** 2022-08-08

**Authors:** Xianqin Yang, Li Ni, Junyu Sun, Xiaolu Yuan, Dezhong Li

**Affiliations:** ^1^ Department of Emergency, Central Hospital of Enshi Tujia and Miao Autonomous Prefecture, Enshi, China; ^2^ Heart Function Examination Room, Wuhan Third Hospital & Tongren Hospital of Wuhan University, Wuhan, China; ^3^ College of Health and Nursing, Wuchang University of Technology, Wuhan, China; ^4^ Department of Pathology, Maoming People’ s Hospital, Maoming, China

**Keywords:** polymorphism, type 2 diabetes mellitus, susceptibility, meta-analysis, FNDC5

## Abstract

**Background:**

FNDC5 is a novel and important player in energy regulation related to glucose metabolism and insulin levels. Thus, it may affect the incidence of type 2 diabetes mellitus (T2DM). Nevertheless, the association between FNDC5 single nucleotide polymorphisms (SNPs) and susceptibility to T2DM remains unclear. The aim of this meta-analysis was to explore whether the SNPs, rs3480 and rs16835198, are associated with the risk of T2DM.

**Methods:**

Studies published before February 1^st^, 2022 were screened to identify the included studies. R software was also applied for calculation of odds ratio (OR), 95% confidence interval (95% CI), heterogeneity, and sensitivity analysis.

**Results:**

Seven studies for rs3480 (involving 5475 patients with T2DM and 4855 healthy controls) and five studies for rs16835198 (involving 4217 patients with T2DM and 4019 healthy controls) were included in this meta-analysis. The results revealed a statistically significant association of rs3480 with T2DM under homozygote (GG vs AA: OR = 1.76, 95% CI = 1.31–2.37, *P* = 0.0002, I^2^ = 59%) genetic model. However, there was no statistically significant correlation between rs16835198 and susceptibility to T2DM under allelic (G vs T: OR = 1.33, 95% CI = 0.94–1.89, *P* = 0.11, I^2^ = 84%), heterozygote (GT vs TT: OR = 1.17, 95% CI = 0.80–1.69, *P* = 0.42, I^2^ = 71%), homozygote (GG vs TT: OR = 1.35, 95% CI = 0.95–1.94, *P* = 0.10, I^2^ = 62%), recessive (GG+GT vs TT: OR = 1.25, 95% CI = 0.88–1.79, *P* = 0.22, I^2^ = 72%), and dominant (GG vs GT+GG: OR = 1.20, 95% CI = 0.96–1.50, *P* = 0.11, I^2^ = 46%) genetic models.

**Conclusions:**

The present meta-analysis revealed that rs3480 in FNDC5 is significantly associated with susceptibility to T2DM, while rs16835198 does not show such an association.

## 1 Introduction

The number of patients with type 2 diabetes mellitus (T2DM) is increasing worldwide, and T2DM has become one of the most serious medical and health issues worldwide (1) . According to the International Diabetes Federation (IDF), the number of diabetes cases worldwide will reach 600 million by 2035 (2). Diabetes may be accompanied by a variety of complications, such as stroke, blindness, kidney failure, and myocardial infarction (3). Furthermore, it should be noted that even in individuals with mild hyperglycemia (prediabetes), such complication had been observed (4–6). These complications are the main cause of death and disability in patients with diabetes (4, 7). T2DM not only seriously affects the quality of life of patients, but also brings heavy economic burden to societies and families. Therefore, early detection of T2DM could have important clinical significance, studying the etiology and pathogenesis of T2DM is of great significance to the survival and development of human beings.

Irisin, a novel intriguing myokine, was recently reported and described by Bostrom et al. Irisin is released upon cleavage of the plasma membrane protein fibronectin type III domain containing protein 5 (FNDC5), whose gene expression is suggested to be driven by muscle-specific transgenic overexpression of the exercise-responsive transcriptional co-activator peroxisome proliferator-activated receptor (PPAR)-γ co-activator-1α (PGC-1α) (5, 8). In an animal model of obesity and T2DM, irisin intervention increases mitochondrial uncoupling, mitochondrial oxidative metabolism, and fatty acid oxidation in skeletal muscle (6, 9). Clinical studies have discovered that there is an association between irisin levels and metabolic disturbance. Its serum concentration is reduced in patients with T2DM, obesity, metabolic syndrome, and nonalcoholic fatty liver disease (7, 8, 10, 11). Furthermore, young male athletes possess higher irisin levels than middle-aged obese women (9, 12). Therefore, FNDC5 is considered an attractive target for metabolic disease.

The incidence of T2DM is closely related to genetic and environmental factors (10, 13). Searching for pathogenic genes involved in T2DM and revealing the pathogenesis of T2DM at the molecular level can provide help for early detection of individuals at high risk of T2DM and prevention of complications. Single nucleotide polymorphism (SNP) refers to polymorphisms in the DNA sequence caused by variations in a single nucleotide at the genomic level. SNPs are commonly inherited in humans, accounting for more than 90% of all known polymorphisms.

Several studies have evaluated the association of SNPs in FNDC5 with susceptibility to T2DM. However, the results are inconsistent. Therefore, the role of these FNDC5 SNPs in the risk of T2DM remains unclear. Here, we conducted a meta-analysis based on the available data to determine whether FNDC5 rs3480 (G>A) and rs16835198 (G>T) SNPs are associated with susceptibility to T2DM.

## 2 Methods

### 2.1 Guideline selection

In order to ensure the transparency and accuracy of the reporting medical research, the present meta-analysis was conducted following the PRISMA guidelines, as they are appropriate for systematic reviews and meta-analyses (14, 15).

### 2.2 Literature search

PubMed, Embase, Cochrane, China National Knowledge Infrastructure, and Chinese BioMedical Literature databases were used to retrieve literatures systematically. The language of the studies was limited to Chinese and English. The search strategy involved the use of the following terms: “FNDC5,” “fibronectin type III domain containing protein 5,” “type 2 Diabetes mellitus,” “T2DM,” “single nucleotide polymorphism,” and “SNP.” Systematic retrieval was conducted until February 1^st^, 2022.

### 2.3 Inclusion criteria

The inclusion criteria were as follows: (1) case-control study on the correlation between the SNPS, rs3480 and rs16835198, and T2DM risk; (2) the diagnosis of T2DM conforms to WHO diagnostic criteria; (3) the study population in the study is consistent with Hardy-Weinberg Equilibrium (HWE); (4) the literature provides genotypic and/or allelic frequencies of the rs3480 and rs16835198 SNPs.

Studies were excluded if one of the following exclusion criteria was fulfilled: (1) no control group; (2) comments, review, abstracts, letters, conference presentations, and studies on animal models; (3) lack of genotypic and/or allelic frequencies of the rs3480 and rs16835198 SNPs. In case of duplicate publications, the study with the largest sample size was included.

### 2.4 Data extraction and quality assessment

Two authors (Yang and Ni) read the titles of the articles independently and assessed the quality of the included articles. In case of any disagreement, a decision was made after discussion. The two authors extracted the following data from all included articles: first author, year of publication, country of participants, number of cases and controls, genotypic distribution in cases and controls, genotyping methods, and HWE. An external referee was invited in case of disagreements not resolved by both investigators.

We applied the Newcastle Ottawa scale (NOS) to evaluate the quality of eligible studies from different aspects: (1) adequate definition of case; (2) representativeness of the cases; (3) selection of controls; (4) definition of controls; (5) comparability of cases and controls; (6) ascertainment of exposure; (7) same method of ascertainment for cases and controls; (8) non-response rate. The NOS has a score range of 0 to 9, and ≥7 was considered of high quality (14, 16).

### 2.5 Statistical analysis

We employed R (version 4.0.3) software and meta package for statistical analyses. To evaluate the strength of correlation between rs3480, rs16835198, and T2DM under five genetic models, odds ratios (ORs) and 95% confidence interval (CIs) were calculated. Statistical significance was set at *P* < 0.05. Q test and I^2^ statistic were used to assess heterogeneity among the included studies. The heterogeneity was obvious if the *P* value of the Q test < 0.1 or I^2^ ≥ 50% (16, 17).

The random-effect model was used when significant heterogeneity was present, otherwise, the Mantel-Haenszel fixed-effect model was used. Actually, considering the clinical heterogeneity among the observational studies (e.g, sex, age, adjusted confounders, and so on), it would be more proper to use random-effects model first, even not statistical heterogeneity was observed (18, 19), therefore, we used random-effects model to calculate all the genetic models. Sensitivity analysis, test the stability of results, was conducted using R software (4.0.3) and meta package. The publication bias was assessed by *Egger*’s test (17, 20).

## 3 Results

### 3.1 Characteristics of the included studies

**Figure 1:**
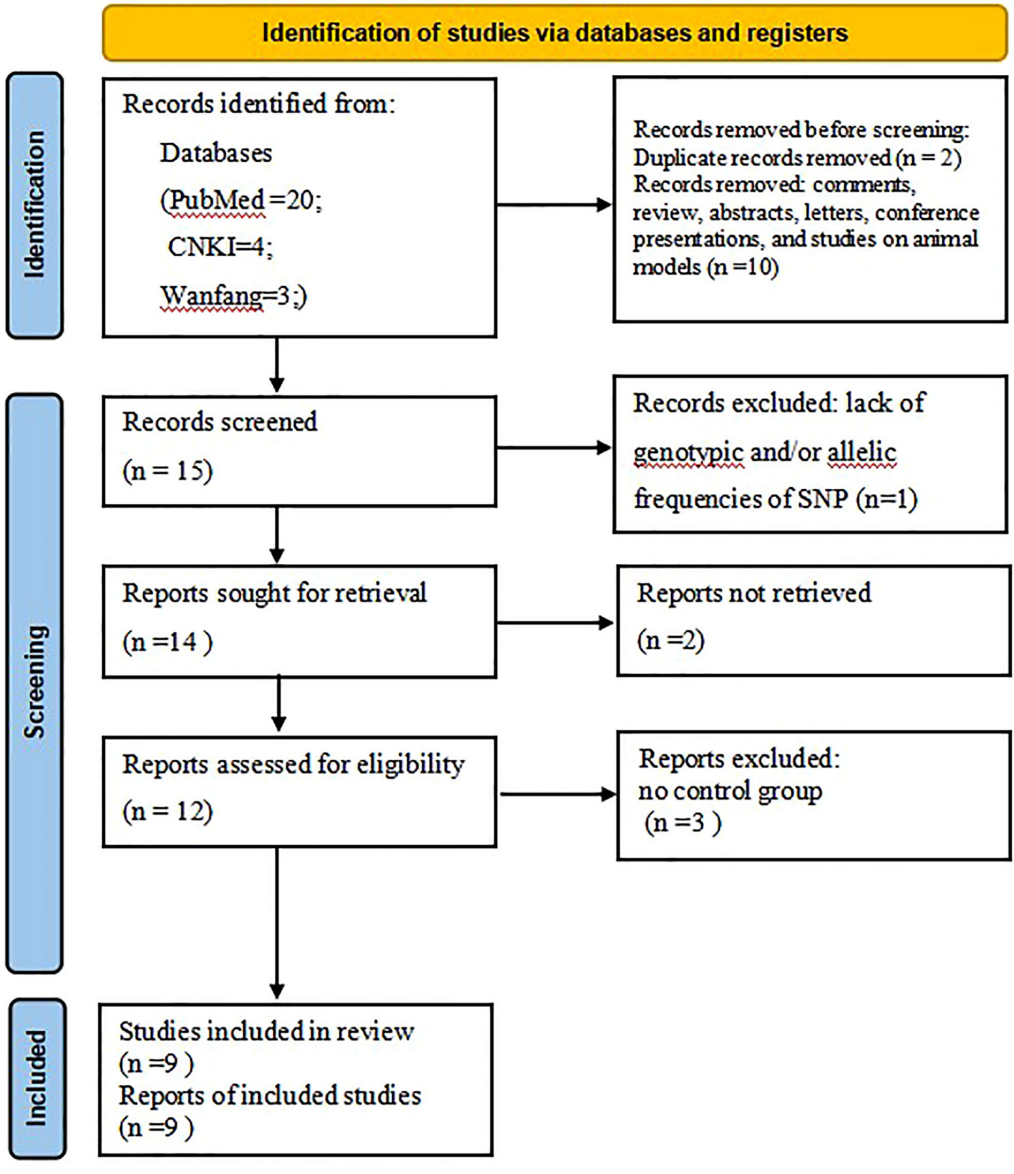
The Flow chart showing the study selection process.

Literature search was carried out according to the PRISMA flow chart shown in **Figure 1**. A total of 27 potentially relevant articles were found after the retrieval process. 15 articles were selected for further analyses after exclusion of all duplicate articles identified by screening through the titles and abstracts. Another 12 articles were subsequently excluded after careful reading of the abstracts and titles. 9 articles were finally included in the present meta-analysis (21–29). **Table 1** shows the qualities of all included studies as determined by NOS evaluation (30). Detailed information of the 9 included articles is presented in **Table 2**.

**Table 1:** The newcastle-ottawa quality assessment scale.

Author (year)	Selection				Comparability	Exposure			
	Adequate definition of case	Representativeness of the cases	Selection of controls	Definition of controls	Comparability of cases and controls	Ascertainment of exposure	Same method of ascertainment for cases and controls	Nonresponse rate	Total score
Tanisawa (29)	*	*		*	**	*	*	*	8
Brondani (23)	*	*	*	*	**	*	*	*	9
Gao (24)	*	*		*	*	*	*	*	7
Tang (21)	*	*		*	**	*	*	*	8
Al-Daghri (25)	*	*		*	**	*	*	*	8
Allah (26)	*	*		*	**	*	*	*	8
Khidr (22)	*	*		*	**	*	*	*	8
Pan (27)		*		*	*		*	*	5
Zabibah (28)	*	*		*	**	*	*	*	8

*Represents one scores following the Newcastle-Ottawa. Quality Assessment Scale. **Represents two scores following the Newcastle-Ottawa Quality Assessment Scale.

**Table 2:** Characteristics of included studies.

Author(year)	Country	Case/Control	Genotype distribution						Genotyping methods	HWE
			Case			Control				
			**rs3480**							
			GG	AG	AA	GG	AG	AA		
Brondani (23)	Southern Brazilian	942/434	178	359	405	79	185	170	TaqMan assay	>0.05
Gao (24)	China	281/286	23	110	148	22	114	150	Mass array	>0.05
Tang (21)	China	3397/3405	267	1275	1855	225	1306	1874	Mass array	>0.05
Al-Daghri (25)	Saudi	376/410	78	181	117	88	186	136	TaqMan assay	>0.05
Allah (26)	Egypt	71/70	24	35	12	10	28	32	TaqMan assay	>0.05
Pan (27)	China	358/200	30	133	195	13	76	111	Mass array	>0.05
Zabibah (28)	Iraq	50/50	7	25	18	4	18	28	PCR- RFLP	>0.05
			**rs16835198**							
			GG	GT	TT	GG	GT	TT		
Tanisawa (29)	Japan	82/81	32	35	15	19	50	12	TaqMan assay	>0.05
Gao (24)	China	280/286	83	150	47	81	131	74	Mass array	>0.05
Tang (21)	China	3397/3402	929	1661	807	899	1735	768	Mass array	>0.05
Khidr (22)	Egypt	100/50	54	37	9	18	21	11	TaqMan assay	>0.05
Pan (27)	China	358/200	97	178	83	50	96	54	TaqMan assay	>0.05

### 3.2 Quantitative analysis

#### 3.2.1 Association between rs3480 and susceptibility to T2DM

Seven studies involving 5475 patients with T2DM and 4855 healthy controls were included in the meta-analysis to explore the potential association between rs3480 and susceptibility to T2DM.

**Figure 2:**
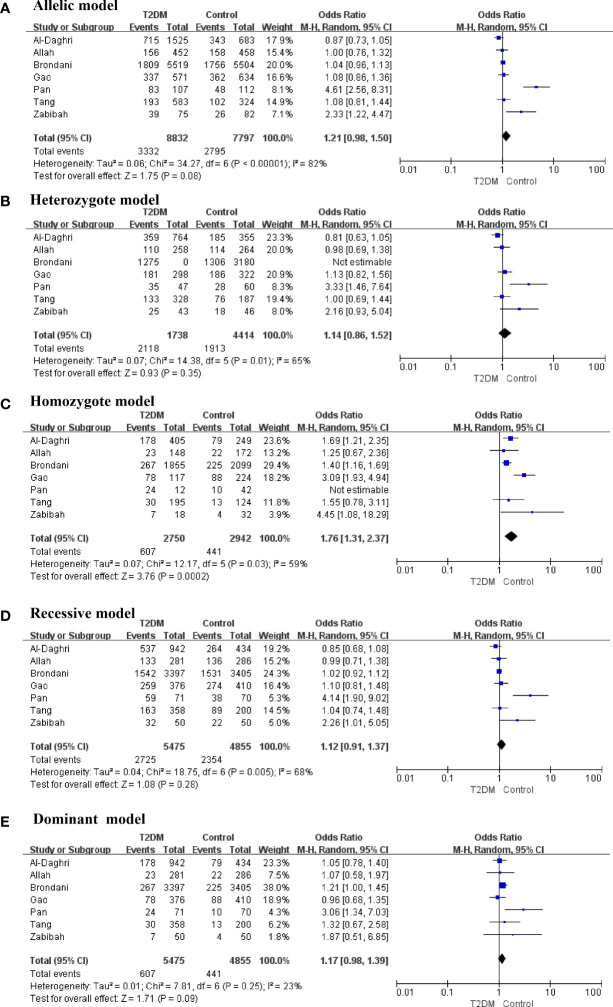
Forest plots of the polymorphism of rs3480 and the T2DM risk under five genetic models **(A-E)**.

The present meta-analysis discovered that the correlations between an FNDC5 rs3480 (G/A) and susceptibility to T2DM in homozygote (GG vs AA: OR = 1.76, 95% CI = 1.31–2.37, *P* = 0.0002, I^2^ = 59%) genetic model was statistically significant. In contrast, no statistical significance was found for correlations between rs3480 and T2DM susceptibility in allelic (G vs A: OR = 1.21, 95% CI = 0.98-1.50, *P* = 0.08, I^2^ = 82%), heterozygote (GA *vs* AA: OR = 1.14, 95% CI = 0.86–1.52, *P* = 0.35, I^2^ = 65%), recessive (GG vs GA+AA: OR = 1.12, 95% C = 0.91-1.37, *P* = 0.28, I^2^ = 68%), and dominant (GG+GA vs AA: OR = 1.17, 95% CI = 0.98–1.39, *P* = 0.09, I^2^ = 23%) genetic models. Our results suggested that people carrying the G allele in rs3480 had higher susceptibility to T2DM. The forest plots are presented in **Figure 2**.

**Figure 3:**
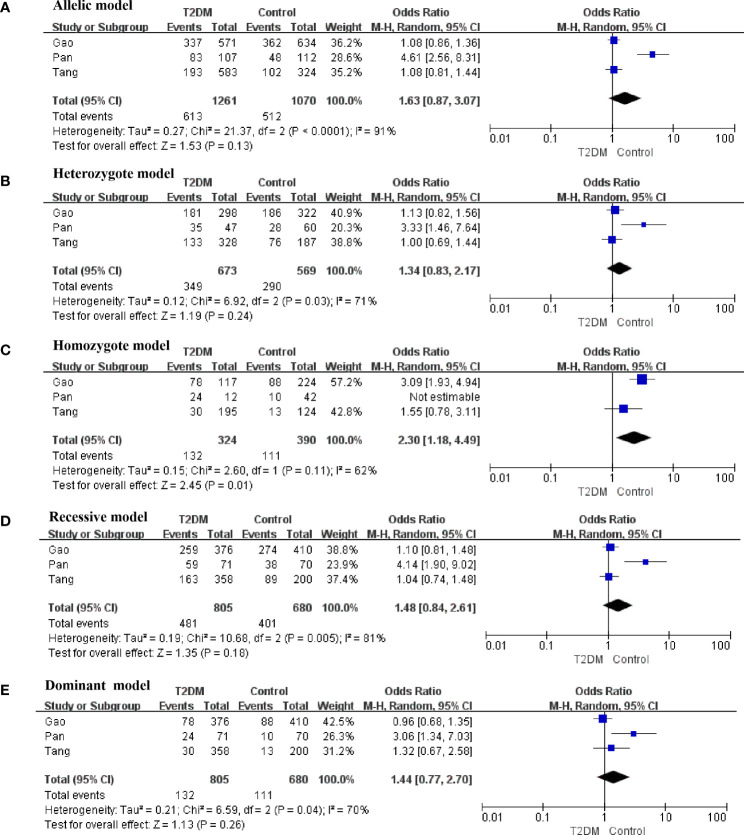
Forest plots of the polymorphism of rs3480 and the T2DM risk under five genetic models in Chinese. **(A)** allelic, **(B)** heterozygote, **(C)** homozygote, **(D)** recessive, and **(E)** dominant genetic models.

**Figure 4:**
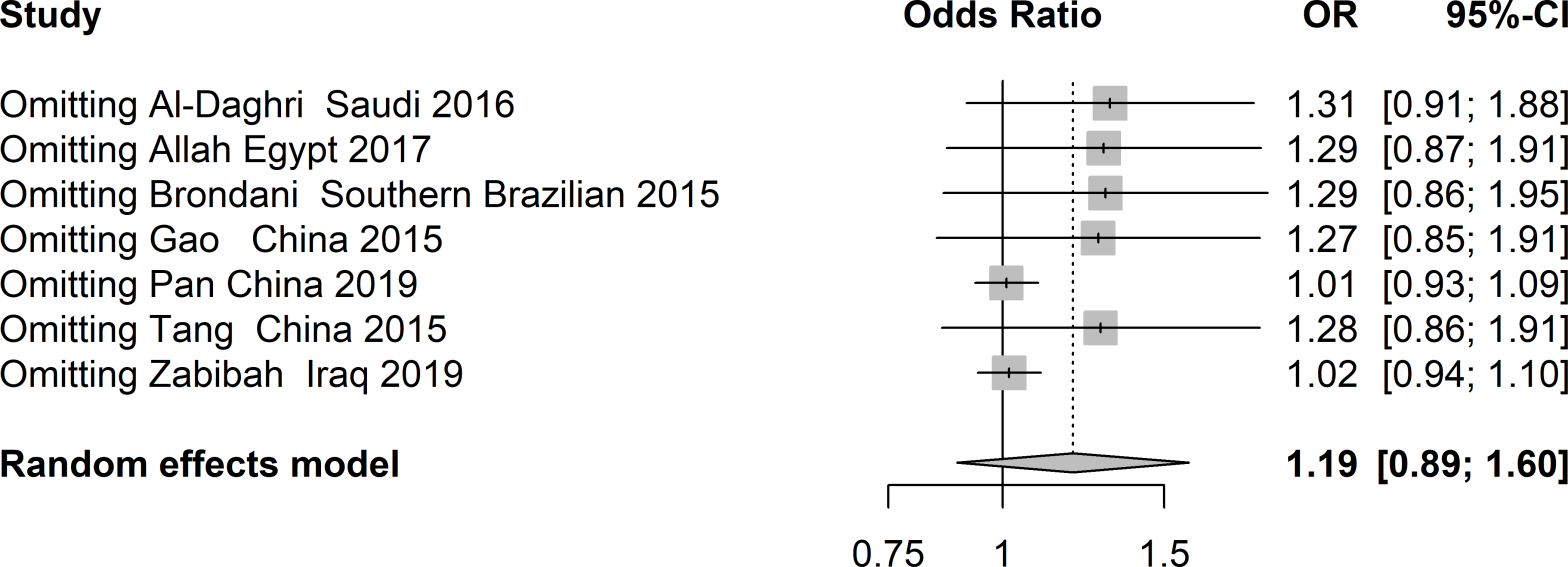
Sensitivity analysis of studies included in analysis of rs3480 and T2DM risk.

There was obvious heterogeneity in the allelic, heterozygote, homozygote, and recessive models; therefore, subgroup analysis was performed. As shown in **Figure 3**, rs3480 possessed a significant association with susceptibility to T2DM in Chinese individuals under the homozygote (GG vs AA: OR = 2.30, 95% CI = 1.18-4.49, *P* = 0.01, I^2^ = 62%) models. Sensitivity analyses were further applied for different genetic models. As shown in **Figure 4**, no significant heterogeneity was observed in any of the genetic models after excluding each study.

#### 3.2.2 Association between rs16835198 and T2DM risk

**Figure 5:**
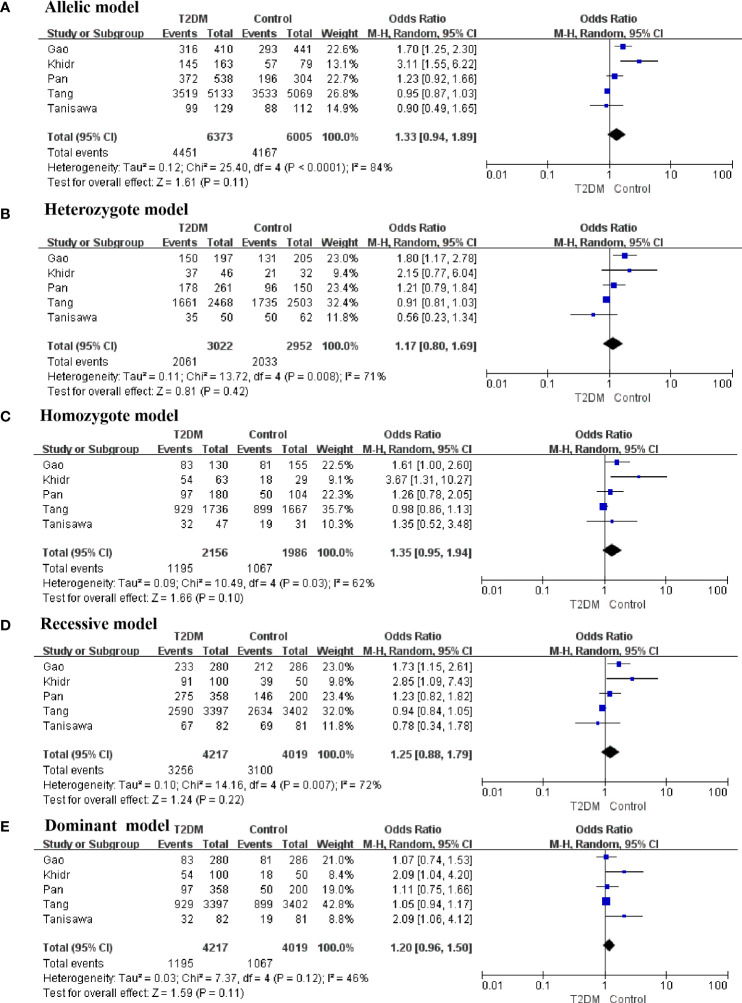
Forest plots of the polymorphism of rs16835198 and the T2DM risk under five genetic models. **(A)** allelic, **(B)** heterozygote, **(C)** homozygote, **(D)** recessive, and **(E)** dominant genetic models.

**Figure 6:**
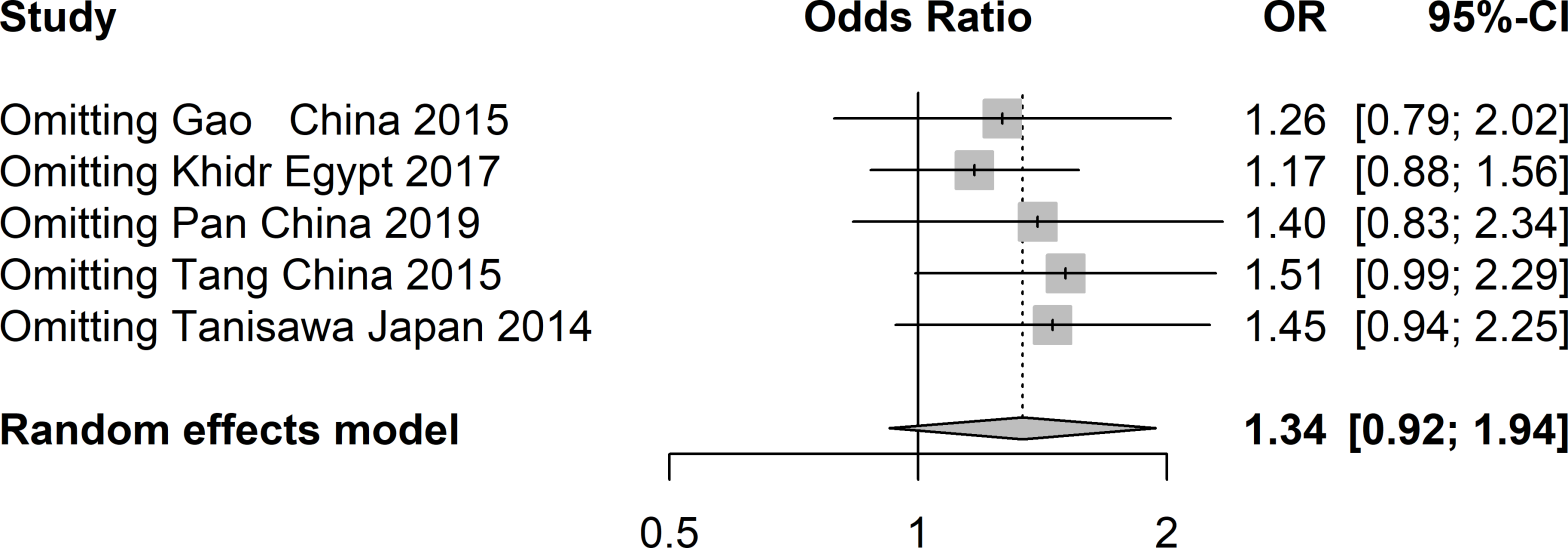
Sensitivity analysis of studies included in analysis of rs16835198 and T2DM risk.

A total of five studies involving 4217 patients with T2DM and 4019 healthy controls were finally included to assess the potential correlation between rs16835198 and susceptibility to T2DM. **Figure 5** showed that there was no association between rs16835198 and susceptibility to T2DM under allelic (G vs T: OR = 1.33, 95% CI = 0.94–1.89, *P* = 0.11, I^2^ = 84%), heterozygote (GT vs TT: OR = 1.17, 95% CI = 0.80–1.69, *P* = 0.42, I^2^ = 71%), homozygote (GG vs TT: OR = 1.35, 95% CI = 0.95–1.94, *P* = 0.10, I^2^ = 62%), recessive (GG+GT vs TT: OR = 1.25, 95% CI = 0.88–1.79, *P* = 0.22, I^2^ = 72%), and dominant (GG vs GT+GG: OR = 1.20, 95% CI = 0.96–1.50, *P* = 0.11, I^2^ = 46%) genetic models. **Figure 6** exhibited the results of sensitivity analyses for the included studies, there was no heterogeneity for all the genetics models after excluding each study.

No obvious asymmetry was observed in the Egger’s test for any comparison, which suggested that the findings were unlikely to be influenced by publication bias. The results of rs3480 and rs16835198 were summarized in **Table 3**.

**Table 3:** Summary OR and 95% CI of rs3480, rs16835198 polymorphisms and T2DM.

SNP	Genetic models	n	OR	95% CI	*P*value	I^2^	*P* publication bias(Egger)
rs3480							
Allelic model	G *vs* A	7	1.21	0.98-1.50	0.08	82%	0.146
Heterozygote model	GA *vs* AA	7	1.14	0.86-1.52	0.35	65%	0.624
Homozygote model	GG *vs* AA	7	**1.76**	**1.31-2.37**	0.0002^*^	59%	0.229
Dominant model	GG+GA *vs* AA	7	1.17	0.98–1.39	0.09	23%	0.402
Recessive model	GG *vs* GA+AA	7	1.12	0.91-1.37	0.28	68%	0.586
							
rs16835198							
Allelic model	G *vs* T	5	1.33	0.94-1.89	0.11	84%	0.620
Heterozygote model	GT *vs* TT	5	1.17	0.80-1.69	0.42	71%	0.098
Homozygote model	GG *vs* TT	5	1.35	0.95-1.94	0.10	62%	0.610
Dominant model	GG+GT *vs* TT	5	1.20	0.96-1.50	0.11	46%	0.384
Recessive model	GG *vs* GT+GG	5	1.25	0.88-1.79	0.22	72%	0.810

Bold values represents significant association.

## 4 Discussion

T2DM is a complex polygenic metabolic disease caused by the interaction of genetic and environmental factors. Unhealthy lifestyles increase the risk of T2DM, but not all individuals with unhealthy lifestyle habits develop the disease. Therefore, genetic factors play a very important role in the onset and progression of T2DM, which needs to be further studied. SNPs are polymorphisms of the DNA sequence caused by variations in a single nucleotide at the genomic level. They are commonly inherited in humans, accounting for more than 90% of all known polymorphisms.

In 2007, Sladek et al. used Genome Wide Association Study (GWAS) to identify diabetes susceptibility genes in the French population. Several research groups further identified and confirmed SNPs associated with diabetes susceptibility. Thus, the association between SNPs and T2DM susceptibility is being gradually revealed (31–33). FNDC5 is a precursor of irisin and can significantly disrupt metabolism. In an obese mouse model, overexpression of FNDC5 enhances energy expenditure, lipolysis, and insulin sensitivity, and improves hyperlipidemia, hyperglycemia, and hyperinsulinemia (15). A high-fat diet increases the mRNA and protein levels of FNDC5 in muscle tissue of obese mice (34). Moreover, FNDC5 protein levels are increased in muscle tissue after endurance training.

Multiple SNPs significantly associated with metabolic disease susceptibility in different populations have been found in the FNDC5 gene. Rs16835198 was found to be significantly associated with insulin sensitivity and obesity in the German and Egyptian populations, respectively (35). The results showed a significant association of the rs16835198 G allele with fasting insulin levels and body mass index in 6822 Chinese Han individuals (21). The G allele of rs3480 has been associated with elevated hemoglobin a1c levels in Brazilian women with T2DM (23). In addition, the rs3480 GG genotype has been associated with a reduced risk of obesity and a lower body weight index in the Saudi population. Therefore, SNPs in FNDC5 are critical for regulating metabolic homeostasis (36). Our meta-analysis showed that rs3480 is associated with susceptibility to T2DM, and that people carrying the G allele have a higher susceptibility to T2DM. Previous studies have shown that miR-135-5p preferentially binds to the G allele of rs3480 after upregulation, thus enhancing the attenuating effect of miR-135-5p on FNDC5 and reducing the FNDC5 mRNA levels, which results in a weakened regulatory effect of FNDC5 on metabolic diseases (37). In addition, miR-135-5p is upregulated in serum and renal tissue of patients with diabetic nephropathy (38). Taken together, these results suggest that the G allele of rs3480 is detrimental to FNDC5 expression, which may explain the association with T2DM.

In addition, our meta-analysis showed that rs16835198 is not associated with susceptibility to T2DM. Rs16835198 is located on the 3’ region of the FNDC5 gene, which is unlikely to affect the amino acid sequence of the protein products (39). Rs16835198 may not be significantly related to FNDC5 expression changes; therefore, rs16835198 is not strongly associated with susceptibility to T2DM. However, the number of articles included in this study is very limited and further exploration is needed.

This meta-analysis has some limitations. First, it included nine articles with large and heterogeneous populations, including three studies on Chinese Han individuals, two on Egyptian populations, and four on individuals from Southern Brazil, Saudi Arabia, Iraq, and Japan each. The differences among races may have affected the results. The best approach would have been to conduct subgroup analysis for each race, but the literature volume of the corresponding subgroups was not sufficiently large. Therefore, a comprehensive analysis can only be conducted after the inclusion of more articles. Second, only English and Chinese articles were included in this meta-analysis, and data presented in other languages were not included.

In conclusion, we found that the rs3480 G allele in FNDC5 may confer moderate risk for T2DM. Further investigation of these SNPs may improve our understanding of the occurrence and progression of T2DM. We are aware that the present meta-analysis results were derived from a limited sample size. Therefore, future analyses with larger sample sizes and including more studies are required to define the associations between rs3480 and T2DM risk.

## Data availability statement

The original contributions presented in the study are included in the article/supplementary material. Further inquiries can be directed to the corresponding author.

## Author contributions

XYa and LN searched literature and collected data. JS and XYu analyzed the data. DL supervised the project. XYa wrote the original manuscript. DL reviewed and revise the manuscript. All authors read and approved the final version of the manuscript.

## Conflict of interest

The authors declare that the research was conducted in the absence of any commercial or financial relationships that could be construed as a potential conflict of interest.

## Publisher’s note

All claims expressed in this article are solely those of the authors and do not necessarily represent those of their affiliated organizations, or those of the publisher, the editors and the reviewers. Any product that may be evaluated in this article, or claim that may be made by its manufacturer, is not guaranteed or endorsed by the publisher.
